# Isolated gastric involvement of recurrent Hodgkin lymphoma: a case report and review of the literature

**DOI:** 10.1186/s13256-023-03918-w

**Published:** 2023-05-05

**Authors:** Soroush Kohansal, Mohammad Ashouri, Narjes Mohammadzadeh

**Affiliations:** 1 Shahed University, Tehran, Iran; 2 Department of Surgery, Imam Khomeini Hospital, Tehran University of Medical Sciences, Tehran, Iran

**Keywords:** Hodgkin lymphoma, Gastrointestinal Hodgkin lymphoma, Gastrointestinal bleeding

## Abstract

**Background:**

Although the gastrointestinal tract is one of the most common sites for extranodal lymphoma, gastrointestinal lymphoma is a rare disease that is almost exclusively non-Hodgkin lymphoma.

**Case presentation:**

We present a rare condition of Hodgkin lymphoma relapse after 12 years as isolated gastric involvement caused massive gastrointestinal bleeding in a 34-year-old Iranian woman. According to the result of the upper endoscopy, laparotomy was performed, and a large mass in the upper part of the fundus, alongside the esophagogastric junction, was seen, so gastrectomy was performed.

**Conclusion:**

All symptoms and paraclinical findings for gastrointestinal Hodgkin lymphoma are nonspecific. Therefore, the preoperative diagnosis is challenging. It seems that surgery is a suitable diagnostic and therapeutic method in this field.

## Background

Lymphomas represent a variety of disorders caused by the clonal propagation of lymphocytes [1]. Lymphoma is classified into two types: Hodgkin lymphoma (HL), which is indicated by Reed-Sternberg (RS) cells, and non-Hodgkin lymphoma (NHL), which is more prevalent [1, 2]. Extranodal lymphoma (ENL) is defined as the discontinuous infiltration of malignant lymphomatous cells in extra-lymphatic organs [3]. ENL is classified into two types: primary and secondary (or relapse). Secondary ENL also includes multi-organ involvement as well as isolated organ involvement [4]. Bones (30.76%), lungs (19.2%), spleen (19.2%), and liver (11.5%) are the most common sites of HL extranodal infiltration [3]. Although the gastrointestinal tract (GI) is one of the most common sites for ENL, GI lymphoma is a rare disease that is almost exclusively associated with NHL [5]. In this case report, we present a rare condition of HL relapse after 12 years caused by isolated gastric involvement that caused massive GI bleeding in a 34-year-old woman.

## Case presentation

A 34-year-old Iranian woman who had five episodes of upper gastrointestinal bleeding (UGIB) in a week, the last of which was substantial, was taken to the emergency room. Throughout the past 6 months, she had lost weight and felt generally weak. Her past medical history included chemotherapy for primary HL with mediastinal lymph node involvement 12 years prior. She did not smoke, drink alcohol, use drugs, take any particular medications, have any known food or medication sensitivities, or have any significant family or social history.

Upon arrival, she was pale and ill. Her vital signs were 90/55 mmHg blood pressure, 112 beats per minute heart rate, 37 °C body temperature, 96% oxygen saturation, and an 18 beats per minute respiration rate. The abdominal examination revealed mild epigastric tenderness without rebound or guarding. Peripheral pulses were weak. The physical and neurological exams turned up no other noteworthy results. Hemoglobin was 4.4 g/dL according to laboratory tests (Table 1).

**Table 1:** Laboratory tests

Test	Result	Unit	Normal	Test	Result	Unit	Normal
RBC	1.52	M/mm^3^	4.2–5.8 M/mm^3^	Ferritin	157	ng/dL	10–24 ng/dL
Hct	13	%	36–51%	ESR	105	mm/H	< 20 mm/H
HB	4.4	g/dL	12–16 g/dL	CRP	115	mg/L	< 6.0 mg/L
MCV	85.5	fL	77–94 fL	ALP	324	U/L	70–360 U/L
WBC	8.44	10^3^/mm^3^	4.1–10.1 10^3^/mm^3^	ALT	11	U/L	< 31 U/L
PLT	187	10^3^/mm^3^	150–400 10^3^/mm^3^	AST	18	U/L	< 31 U/L
PT	13	seconds	11–15 seconds	Total bilirubin	1	mg/dL	0.1–1.2 mg/dL
PTT	27	seconds	25–45 seconds	Direct bilirubin	0.6	mg/dL	< 0.3 mg/dL

RBC: red blood cells, HCT: hematocrit, HB: hemoglobine, MCV: mean corpuscular volume, WBD: white blood cells, PLT: platelets, PT: prothrombin time, PTT: partial thromboplastin time, AST: aspartataminotransferase, ALT: alanine transaminase, ALP: alkalin phosphatase, CRP: C-reactive protein, ESR: erythrocyte sedimentation rate

**Fig. 1:**
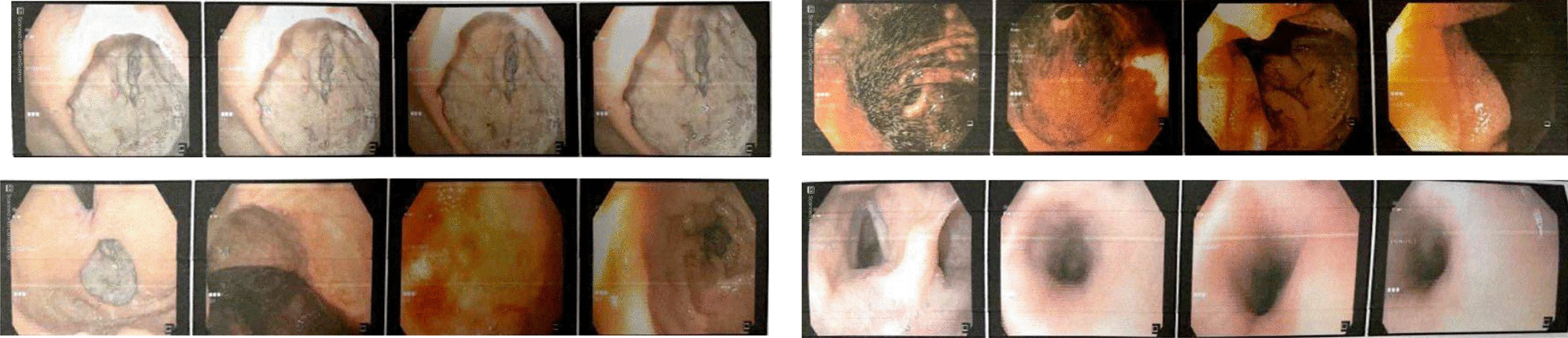
Upper endoscopy images. Endoscopic view of the stomach mass where the tumor is visible from inside the stomach

Monitoring, resuscitation with intravenous fluid, packed cells, and pantoprazole infusion were the first steps in the treatment plan. A big penetrating and necrotizing stomach ulcer with four vessels and no current bleeding was discovered during the patient’s upper GI endoscopy. Endoscopic therapy or biopsy was not feasible due to the substantial risk of bleeding (Fig. 1).

**Fig. 2:**
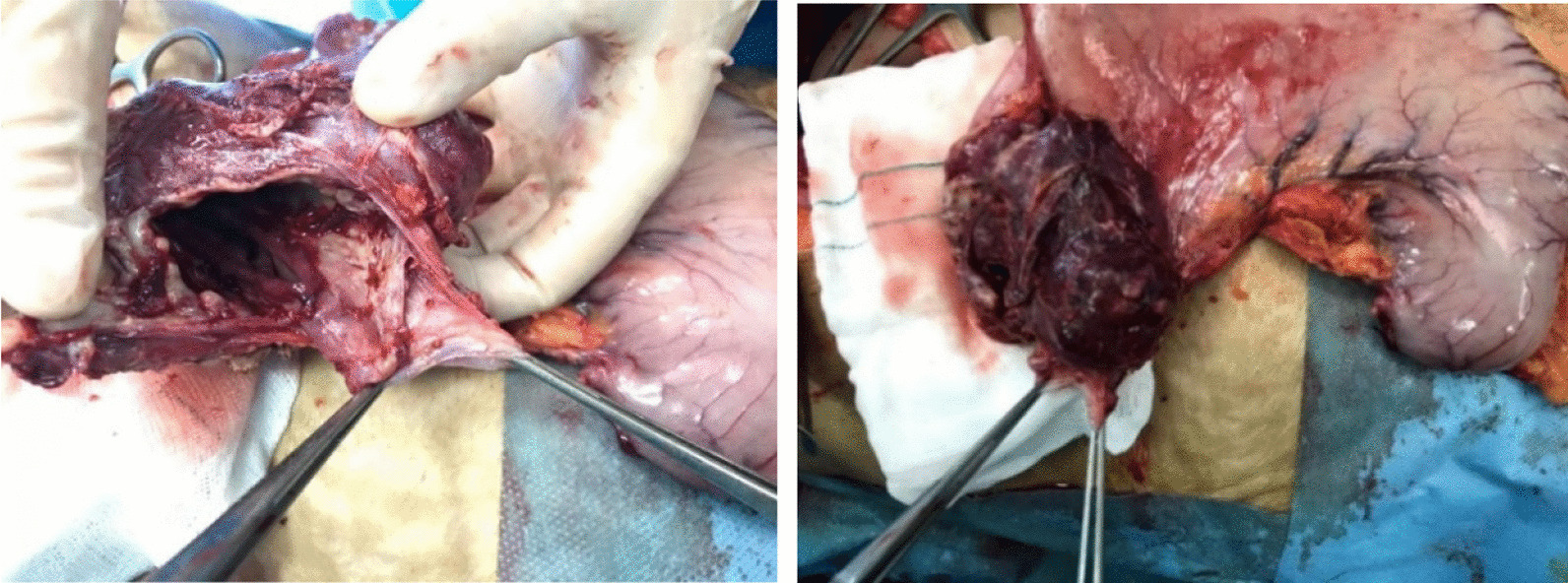
Surgically excised stomach. The image of the stomach excited by gastrectomy can be seen. In the image, a 4-cm opening of the tumor can be seen

**Fig. 3:**
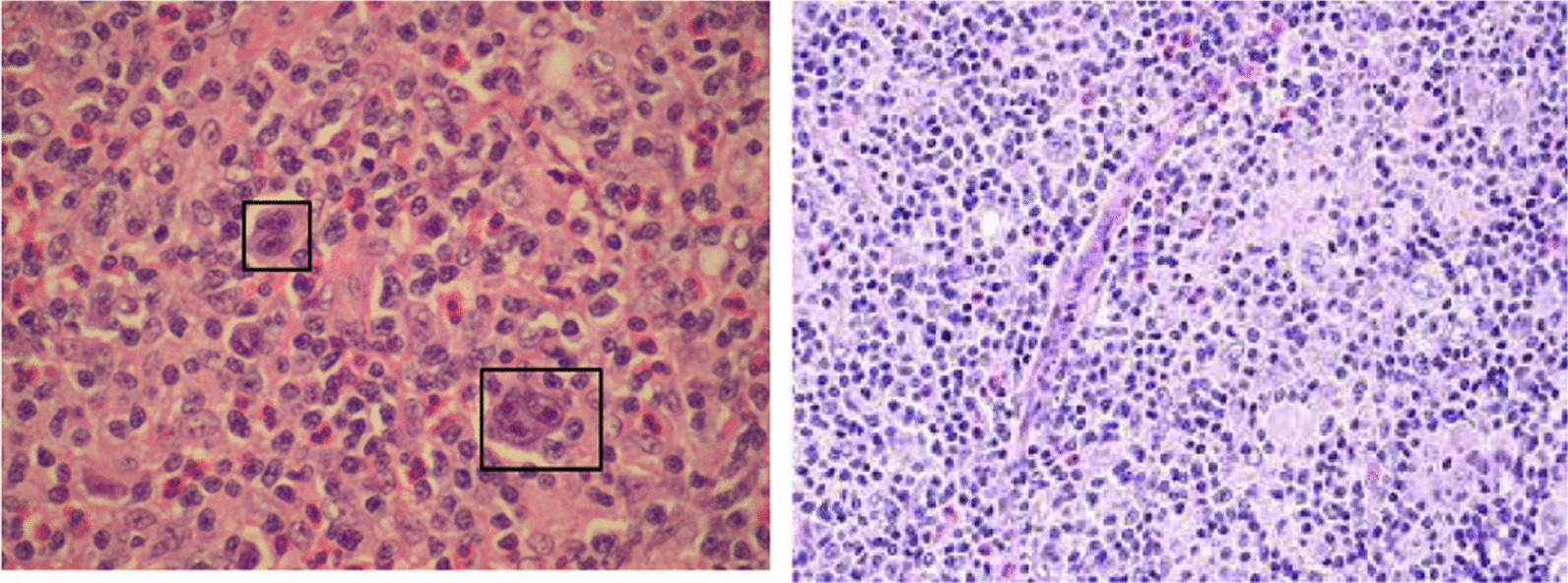
Histopathological pictures suggesting HL [6]. Reed-Sternberg cells are shown in the left picture

After receiving a transfusion of 3 units of cross-matched packed cells, the patient was sent to the operating room 12 hours after being admitted. A midline laparotomy was performed, and the abdomen was free of seeding and ascites. The stomach was distended and clotted. A big mass was seen in the upper section of the fundus, near the esophagogastric junction. The tumor’s superior face was punctured but sealed by the diaphragm. In addition, the patient showed a 17.5 cm-long obvious splenomegaly. The procedure involved a total gastrectomy and reconstruction using a Roux-en-Y esophagojejunostomy anastomosis. Since the esophagus’s thickness was normal, the anastomosis was made at the abdomen’s subdiaphragmatic area. Images of the surgically excised stomach are shown in Fig. 2. She moved to the intensive care unit where she would remain until full recovery. She was discharged on the tenth postoperative day after regaining feeding tolerance and bowel habits. Pathology reports revealed that the patient had HL (Table 2, Fig. 3). The patient was requested to return to the clinic 2 weeks following surgery for an early postoperative follow-up, at which she had acceptable vital signs, good condition, and food tolerance. The patient was scheduled for an oncology consultation. An abdominal computed tomography (CT) scan with intravenous contrast was ordered for her, and it revealed no evidence of recurrence. The oncologist began a four-cycle chemotherapy course using an adriamycin, bleomycin sulfate, vinblastine sulfate, and dacarbazine (ABVD) regimen.

**Table 2:** Pathologic report

Description	Diagnosis
Tumor characteristics: 1. Tumor site: Fundus 2. Tumor relationship to GEJ>: The tumor is located in the proximal stomach, and the tumor midpoint is 2 cm or more from the gastroesophageal junction 3. Tumor size: 9 × 8 x 4 cm 4. Distance of tumor midpoint from the gastroesophageal junction: 1.5 cm 5. Tumor configuration: depressed ulcerated Distances of tumor from: proximal margin, 2 cm; distal margin, 13 cm; lesser omental margins, 5 cm; and greater omental margin, 10 cm	Stomach, total gastrectomy: -Histologic type: Hodgkin lymphoma -Histologic grade: classic type -Tumor site: fundus -Tumor size: 9 × 8 x 4 cm -Tumor extension: visceral peritoneum -All surgical margins are free -Distance of the tumor from the closest (proximal) margin is 2 cm -Regional lymph nodes: * Number of lymph nodes examined: 10 * Number of lymph nodes involved: 3 (CD30 is positive in rare RS cells) -Omentum: uninvolved by tumor -pTNM stage classification (AJCC 8th Edition): pT4 pN2

GEJ: gastroesophageal junction, CD: cluster of differentiation, AJCC: american joint committee on cancer

## Discussion and conclusion

Although HL with particular stomach involvement is uncommon, cases have been reported in the literature, which we shall discuss below. The recurrence of this situation is what distinguishes our case.

Although massive UGIB is a life-threatening condition with several differential diagnoses, it has specific diagnostic and therapeutic criteria that include pre-endoscopic care, endoscopic intervention, and post-endoscopic therapy. First, the patient should be hemodynamically stable, as we ensured in our patient. Urgent endoscopy for both diagnostic and therapeutic purposes should be performed within 12 hours in patients with high-risk clinical features, such as in our case [7]. Surgery is recommended when endoscopic treatment is not possible. On the other hand, patients with extensive UGIB caused by malignant lesions, such as in our case, respond poorly to endoscopic therapy [8].

HL is a treatable malignant nodal condition. Extranodal involvement in HL is substantially less prevalent than in NHL. Even when extranodal involvement is present in HL, a related diffusion pattern is usually visible [9]. ENL in the GI tract affects 10–30% of all NHL patients. The most common location for extranodal NHL is the stomach. The small bowel, throat, big bowel, and esophagus are listed next. HL seldom involves GI [10]. In 1981, Jorke *et al*. documented 100 patients with malignant lymphoma and discovered 7 with primary GI involvement and 2 with secondary forms. Two patients had HL, and seven had NHL [11].

Esophageal HL is a rarely reported condition that almost exclusively is secondary to the stomach, adjunct mediastinal lymph node, or cervical lymph node [12]. The patient’s common symptoms are dysphagia, weight loss, dyspepsia, or UGIB [13]. To ascertain the anatomical specifications, radiologic characteristics at the barium examination are useful [9]. Nodular, polypoidal, ulcerated, or stenotic appearances are common endoscopic findings [13]. Surks *et al*. examined known instances of esophageal HL up to 1996. Sixteen patients, or 53%, were diagnosed during the postmortem investigation. Others displayed dysphagia. Ten patients (33%) had HL, like our case, and four patients (14%) had no prior history of the disease [14].

It was thought that HL contained 9% of all gastric lymphoma [9], but with the reclassification of many prior cases of gastric HL to NHL, this incidence decreased to 1% [15]. Patients usually suffer from abdominal pain, nausea, vomiting, and gastrointestinal bleeding (GIB), similar to our case [13]. Endoscopic findings range from nonspecific gastritis to ulcers and tumoral mass lesions, as in our case [15]. Immunohistochemical studies may be confusing, as CD20 may be expressed in classic HL, and CD30-positive cells may be seen often in T-cell lymphoma [16]. Finding Reed-Sternberg cells in a biopsy can provide a definitive diagnosis [2], although doing so will be challenging given their rarity [15]. Zaloznik *et al.*, considered 356 patients with HL in 1992. Only one patient (0.28%) had gastric recurrence without any other evidence of HL, as in our case, and six patients (1.71%) had evidence of disseminated disease with gastric involvement [17].

The ileum is the most common location for intestinal lymphoma. Common symptoms of HL of the small and large intestines are fever, abdominal pain, diarrhea, hematochezia, and weight loss, called "sprue-like syndrome". Colonoscopy and capsule endoscopy can reveal ulcerated lesions or widespread mucosal thickening with a coarse or fine granular appearance. Moreover, an abdominal CT scan can detect mucosal ulcers, blockage, or thickening of the intestinal wall [9, 13]. Hall *et al*. presented a 9-year HD-treated patient who had a second colonic relapse in 1988, cured by postoperative chemotherapy [4].

As we established earlier, all symptoms and paraclinical data for GI HL are nonspecific. Thus, the preoperative diagnosis is problematic. According to Ogawa *et al*., only 3% of gastric HL is appropriately identified before surgery in Japan; it is most commonly misdiagnosed as large B-cell and T-cell NHL [18]. The function of urgent surgery is crucial in GI HL cases that present with complications like UGIB, and according to the nature of the illness, it should be followed by chemotherapy [15].

## Data Availability

Data sharing does not apply to this article as no datasets were generated or analyzed during the current study.

## Abbreviations

HL: Hodgkin lymphoma
NHL: Non-Hodgkin lymphoma
ENL: Extranodal lymphoma
GI: Gastrointestinal
UGIB: Upper gastrointestinal bleeding
